# Flexible motor adjustment of pecking with an artificially extended bill in crows but not in pigeons

**DOI:** 10.1098/rsos.160796

**Published:** 2017-02-15

**Authors:** Hiroshi Matsui, Ei-Ichi Izawa

**Affiliations:** 1 Department of Psychology, Keio University, Tokyo, Japan; 2 Japan Society of Promotion for Sciences, Tokyo, Japan

**Keywords:** birds, pecking, motor adaptation, body mapping, body scheme, tool use

## Abstract

The dextrous foraging skills of primates, including humans, are underpinned by flexible vision-guided control of the arms/hands and even tools as body-part extensions. This capacity involves a visuomotor conversion process that transfers the locations of the hands/arms and a target in retinal coordinates into body coordinates to generate a reaching/grasping movement and to correct online. Similar capacities have evolved in birds, such as tool use in corvids and finches, which represents the flexible motor control of extended body parts. However, the flexibility of avian head-reaching and bill-grasping with body-part extensions remains poorly understood. This study comparatively investigated the flexibility of pecking with an artificially extended bill in crows and pigeons. Pecking performance and kinematics were examined when the bill extension was attached, and after its removal. The bill extension deteriorated pecking in pigeons in both performance and kinematics over 10 days. After the bill removal, pigeons started bill-grasping earlier, indicating motor adaptation to the bill extension. Contrastingly, pecking in crows was deteriorated transiently with the bill extension, but was recovered by adjusting pecking at closer distances, suggesting a quick adjustment to the bill extension. These results indicate flexible visuomotor control to extended body parts in crows but not in pigeons.

## 1. Background

Animal foraging behaviour has evolved in close association with diverse forms of body structures, which founds perception and motor control to find and catch target food resources species-specifically. Dextrous foraging skills of bipedal humans and non-human primates are based on the visually guided control of arms and hands. This capability is based on sensorimotor conversion mechanisms in which visually detected locations of a target (i.e. food) and the arms and hands in retinal coordinates are converted into body coordinates to generate motor commands for reaching and grasping movements. Once generated, the movement is corrected online using visual feedback information of the ongoing arm movement [1–3]. This flexible visual guidance of motor control plays a crucial role in tool manipulation as part of the extended body in humans and non-human primates [4].

Pecking, a typical bird feeding behaviour, is a visually guided behaviour consisting of two motor components, including head-reaching and bill-grasping. Although head-reaching and bill-grasping in birds are considered analogous to arm-reaching and hand-grasping in primates [4], it is unknown whether avian pecking is controlled by similar visuomotor mechanisms as in primate reaching because of the clear anatomical differences between primates and birds. In primates, hands and arms (i.e. effector organs) are anatomically separated from the eyes (i.e. sensory organ) on the head. This primate anatomy enables their eyes to view the locations of a target and their hands in a stable view, and to control the reaching movement using online visual feedback. However, in birds, both the bill and eyes are mounted together on the head, which causes an unstable and moving view associated with the head-reaching movement. This avian anatomy poses the question, what visuomotor mechanism controls avian pecking?

Past research on avian pecking, mostly using pigeons (*Columba livia*) feeding on small seeds and grains on the ground, suggested that pecking is feed-forwardly controlled, but not corrected online using visual feedback signals. Avian pecking is constructed by three movement elements: a fast standstill of head movement in front of a target food item, which is called ‘fixation’, initiation of head-reaching to the target and grasping the target by the bill [5–7]. Once head-reaching has been initiated, pigeons typically close their eyes during pecking, suggesting that vision only plays a role in planning the reaching and grasping movements based on the target location and size determined during fixation, and is not involved after initiation of the movement. Thus, avian pecking is possibly controlled feed-forwardly according to movement preplanned during fixation without online visual feedback for correction [8–11].

However, recent findings on dextrous foraging skills in non-ground feeding birds suggest the active role of vision in flexible motor control. One of the prominent cases is tool use, where the basic motor component is head-reaching. Tool use has been observed in corvids, parrots and finches in the wild and/or laboratory [12–20]. Particularly, in New Caledonian crows (*Corvus moneduloides*; NC crows), the most dextrous tool-using birds in the wild, recent studies of comparative anatomy have revealed morphological foundations of vision facilitation in tool-using behaviour. Specifically, NC crows possess extraordinarily large visual fields in the frontal view [21], and an upturned and stoutly shaped lower bill, which possibly lifts up the bill-held tool into the frontal view position [22]. These morphological features of NC crows could facilitate the use of vision for flexibly controlling the tool as a bill extension.

The active role of vision in flexible motor control based on head-reaching and bill-grasping components was also suggested in trained tool-using behaviour by large-billed crows (*C. macrorhynchos*), which are generalist omnivorous/carnivorous foragers, but non-tool users in the wild [23]. In the previous study, the crows were trained to manipulate an L-shaped rake tool by the bill, holding it to retrieve a food item on a table, and then were tested for the effect of visual blocking on the tool-use trajectory during tool manipulation. Visual blocking with an opaque shield to occlude the tool tip and target food during manipulation deviated the tool manipulation trajectory from that without visual blocking, suggesting the contribution of vision to ongoing tool manipulation. These recent studies in birds suggested that head-reaching and bill-grasping as a basis for motor control of extended body parts are not feed-forwardly controlled, but are more flexible than previously documented in research primarily using pigeons. However, to our knowledge, no empirical study has tested the flexibility of reaching-based pecking control with extended body parts in birds.

To bridge our understanding of flexible pecking control for dextrous foraging skills at the mechanism level in birds, we investigated motor adjustment ability of pecking with artificially extended bills. In this study, we tested crows, in which flexible foraging skills have been documented in the wild and experimental settings [13,14,16–18,23,24], compared with pigeons, in which sensorimotor mechanisms for pecking have been extensively studied in previous research [5–11]. For these two species, the effects of attaching and removing the bill extension were examined in terms of the success rate and kinematics. Herein, we expected three possible effects. First, if the visuomotor mechanism for pecking actively uses ongoing visual information of the bill and target during pecking, the bird would be able to quickly adjust to the bill extension and its removal, and thus, no severe effect would occur after attachment and removal of the artificial bill extension. Second, if the visuomotor control of pecking rarely uses ongoing visual information, pecking would be adjusted through trial-and-error experiences as feedback learning signals to determine the appropriate kinematic parameters. In this case, the bill extension would cause severe effects on both the success rate and kinematics, and its removal would produce a motor after-effect because motor adaptation occurred during the trial-and-error period. Third, if there was no flexibility in visuomotor control to the bill extension, there would be severe effects on both performance and kinematics, but no after-effects would occur.

## 2. Material and methods

### 2.1. Subjects and housing

Three adult pigeons (unknown sex, 290–349 g in body weight) and three sub-adult large-billed crows (three females, 510–715 g body weight) were used in this study. All birds were experimentally naive and wild-caught in Tokyo, as authorized by the Environmental Bureau of Tokyo Metropolitan Government (Permission #4005). Pigeons and crows were kept in different rooms and housed individually in stainless steel-mesh home cages (*W* 35 cm × *D* 30 cm × *H* 35 cm for pigeons, *W* 43 cm × *D* 60 cm × *H* 50 cm for crows) for approximately one month for the experimental period plus 3 days for acclimation to the experimental chamber. Conspecific individuals were placed side-by-side to allow them visual and audio-vocal social communication with one another. During the experimental period, crows were regularly transferred into an outdoor aviary (1.5 m × 2.8 m × *H* 1.7 m) for bathing and direct social interactions with other conspecifics, but without feeding there, for 2–3 h after these daily sessions. After the end of the experiment, birds were transferred back to relatively large outdoor aviaries for group housing (3 m^2^ × *H* 1.5 m for pigeons, 100 m^2^ × *H* 3 m for crows) and used for other behavioural studies. Mixed grains with mineral supplements, and dry foods, cheese and eggs were fed as daily diets for pigeons and crows, respectively. During the experimental period, feeding was controlled with no food 3 h before the daily session, and sufficient food in cups was provided after the session. Water was freely available in the home cages. The room was maintained at 21 ± 2°C in a 13 L : 11 D cycle with light onset at 08.00. The experimental and housing protocols adhered to Japanese National Regulations for Animal Welfare and were approved by the Animal Care and Use Committee of Keio University (no. 14077).

### 2.2. Apparatus

**Figure 1.**
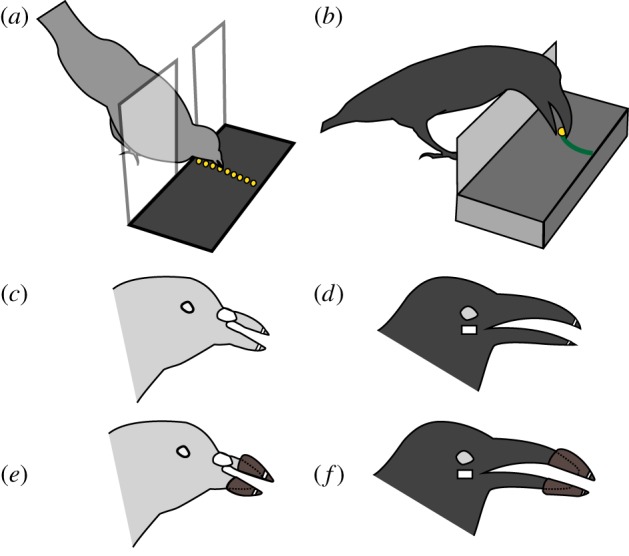
Schematic drawings of the platform table and artificial bill attachment. (*a*) Set-up for pigeons. The sequential pecking array of hempseeds (yellow dots) on the platform table was video-recorded for pigeons. (*b*) Set-up for crows. Pecking a small piece of cheese (yellow dot) on the tip of a wire was video-recorded for crows. (*c*,*d*) Removable tacking markers (white squares) were placed on both the upper and lower tips of the original bills. (*e*) Artificial bill attached onto a pigeon's bill. The attachment extended the bill approximately 1 cm (i.e. 43%) longer than the original length. Tracking markers (white squares) were placed on the tips of the extension. (*f*) A drawing of the artificial bill attached onto a crow's bill. The attachment extended the bill approximately 2 cm (i.e. 31%) longer that the original length. Tracking markers (white squares) were placed on the tips of the extension.

The experiment was conducted in an experimental chamber (*W* 35 cm × *D* 30 cm × *H* 35 cm for pigeons, *W* 60 cm × *L* 63 cm × *H* 45 cm for crows). The chamber consisted of a platform table (*W* 24 cm × *D* 12 cm at 1 cm above the floor for pigeons, *W* 39 cm × *D* 19 cm and 10 cm above the floor for crows) and a stop panel at the front end to prevent the subject from stepping onto the table. The stop panel for pigeons had a 4.5-cm gap in which pigeons could peck seeds on the table through this opening while keeping their body in front of the table (figure 1*a*). The panel for crows was closed and 8 cm high, to prevent crows from pecking straightway along the floor to the target and to facilitate their pecking over the panel along the curved trajectory comparably to the pecking of the pigeons (figure 1*b*). Given the different behavioural patterns of pecking under natural feeding situations, food used as pecking targets was presented on the table in different ways to pigeons and crows in each trial. For pigeons, which typically exhibit sequential pecking of seeds and grains scattered on the ground, an array of 10 hempseeds was presented at 10 specific positions in a line with 1-cm intervals (figure 1*a* and electronic supplementary material, movie P1). For crows, which typically exhibit non-sequential single-shot pecking at a target, a small piece of cheese (approx. less than 1 cm sphere) was presented 10 cm in front of the stop panel and on the tip of a metal wire to lift it 1 cm above the table (figure 1*b*; electronic supplementary material, movie C1). Using these experimental settings, we could video-record the pecking movement of the birds from a sagittal view for the two species for comparison.

### 2.3. Procedures and bill attachment

For both pigeons and crows, the experiment consisted of four phases: *control* (normal bill) phase (1–3 sessions), *bill-extension* phase (10 sessions), *bill-extension removal* phase (1 session) and *follow-up control* phase (1–3 sessions). We conducted one session per day for 20–22 successive days with a one-week interval between the *bill-extension* removal and *follow-up control* phase. Each daily session consisted of five trials for pigeons and 15 trials for crows. In each trial, an array of 10 hempseeds and a small piece of cheese was presented to pigeons and crows, respectively, which allowed us to record 50 pecks from pigeons and 15 pecks from crows in most daily sessions. We tripled the number of pecks for pigeons relative to that of crows because pigeons failed to improve their pecking performance with the bill extension through 10–15 pecks × 10 sessions (Matsui 2016, unpublished thesis). A trial was terminated when the subject consumed the presented food(s) or the subject did not respond to the food within 5 min for both pigeons and crows.

In the control phase, the subjects were given food to peck with normal bills for one to three sessions until 30 trials were completed. On the day following the final session of the control phase, the subjects received a brief surgery (i.e. approx. 10 min) to attach an artificial bill on both the upper and lower bills with super-glue under anaesthesia induced by inhalation of 3% isoflurane (Mylan Inc., Canonsburg, PA, USA). The artificial bill was formed to fit the original bill using dental resin and did not prevent the birds from feeding in the home cage. Artificial upper and lower bills were similarly shaped with the following dimensions: 0.5 ± 0.1 g weight, 1 cm width and 1 cm depth for pigeons and 5.0 ± 1.0 g weight, 2 cm width and 1.5 cm depth for crows (figure 1*e*,*f*). With this attachment, bills were extended approximately 1 and 2 cm past the original bill length of pigeons (mean ± s.e.m. = 2.3 ± 0.1 cm) and crows (6.5 ± 0.1 cm), respectively. Thus, the mean (±s.e.m.) relative length of bill extension to the original bill was 1.43 ± 0.02 and 1.31 ± 0.01 for pigeons and crows, respectively. The shape of the artificial bill was formed to fit the bill shape of each species, but was as similar as possible to avoid an advantage/disadvantage for pecking between the species. It was noted that both pigeons and crows exhibited their response in the experimental session after the surgery without any disturbance of general behaviour, such as non-specific effects caused by the handling and surgery *per se*.

Four hours after confirming recovery from the surgery, the bill-extension phase was begun for 10 sessions. Thereafter, the artificial bill was removed using glue remover, which was accomplished quickly without anaesthesia. Immediately after the removal, the subjects were tested in one session that was the bill-extension removal phase to assess the instantaneous effect of bill removal. If the motor learning (i.e. motor adaptation) to adjust to the bill extension occurred during the 10 sessions with the bill extension, the adjusted motor pattern would be observed immediately after the removal as a remaining effect, a ‘motor after-effect’.

One week after the bill-extension removal session, 30 pecks were recorded from each subject during 1–3 sessions as a follow-up control phase to examine the recovery of pecking movement, and if pecking had adjusted to the bill extension by motor learning, to determine if a similar level as that in the initial control phase was regained.

### 2.4. Video recording and tracking of pecking movements

Pecking movements were video-recorded with a high-speed camcorder (300 frames s^−1^, Gig-E 200, Library Inc., Tokyo, Japan) placed on either of left or right side of the experimental chamber. To measure the kinematic parameters of pecking movement, coordinates of tracking markers on anatomical landmarks of the head and bill were extracted frame-by-frame from sagittal-view two-dimensional images offline using video-tracking software (Move-tr/2D v. 7.0, Library Inc., Tokyo, Japan). Removable white markers (less than 1 cm^2^ square) were attached to the tips of the upper and lower bills for tracking bill-grasping movements (figure 1*c*–*f*). The markers were placed on the tips of the original bills without the extension (i.e. during control, removal and follow-up phases; figure 1*c*,*d*), and on the extended bills when present (i.e. bill-extension phase; figure 1*e*,*f*). For crows, an additional marker was placed below the right eye for tracking head-reaching movements (figure 1*d*,*f*). No tracking marker was attached on the head of pigeons because their white nose knobs were used for tracking head movements.

### 2.5. Analysis

We considered a single pecking action as a movement sequence from the ‘head fixation’, which was characterized by a rapid standstill of the head in front of a target food, to a ‘grasping offset’, which was defined as the timing of the minimal aperture. To examine the effect of the bill extension on feeding performance, we compared the rate of success of food ingestion among the sessions using generalized linear mixed models with a binomial error distribution and a logit link function. The models included the pecking outcome (success or failure) as a response variable, the session as an explanatory variable and the individual as a random factor. If the model analyses showed a significant effect for the session variable, 95% confidence intervals were compared between the control and each of the other sessions to determine the affected sessions. Overdispersion of the model was checked by a dispersion parameter, which was defined as the residual divided by the residual degrees of freedom [25].

**Figure 2.**
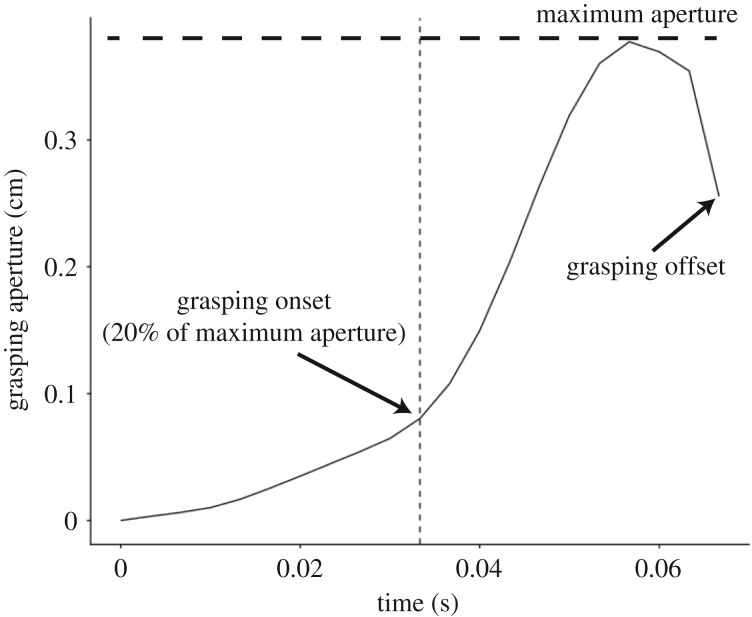
A diagram representing the size of bill aperture along the time course of a peck. Aperture size was measured as the distance between the marker-tracked tips of the upper and lower bills. Note that the size of grasping offset was not zero because of the size of ingested food. This instance was drawn from a pigeon in the control phase.

For kinematic analysis, we extracted coordinates of tracking markers across the sequential video-captured images of each pecking action. The extracted coordinates, after smoothing via 5 Hz Butterworth low-pass filtering, were used to measure several kinematic parameters, including the timing of grasping onset, head-reaching movement distance, mean head-reaching velocity and mean head-reaching acceleration for each pecking event. Grasping onset was defined as a 20% opening of the maximum grasping aperture, which was measured as the maximum distance between the marker positions of original or extended upper and lower bills in each pecking event (figure 2). The movement distance was calculated as the total length of the movement trajectory of the head for each peck. The mean head-reaching velocity was calculated as the average of instantaneous velocities, each of which was calculated by two subsequent frames, from head fixation to grasping onset in each pecking event. The mean head-reaching acceleration was calculated as the average of instantaneous acceleration, each of which was calculated by two subsequent velocities, from fixation to grasping onset in each pecking event. Exceptional instances, such as tracking markers out of the video-frame because of head rotation or neck twisting, were discarded from the analysis.

For the analyses, we hypothesized that bill extensions would cause a certain temporal shift in adjustment in the pecking action because the bill extension should shorten the time for the bill tip to contact a target food compared to pecking with normal bills; thus, birds needed to adapt to this temporal effect with a certain kinematic change, such as opening extended bills earlier than with normal bills to compensate for the shortened time to contact with the target. If this hypothesis is true, the timing of grasping onset from head fixation would be earlier after the attachment of bill extension than before its attachment. To assess this hypothesis, we executed linear mixed models, which consisted of the timing of grasping onset from head fixation as a dependent variable, the experimental phase, movement distance, and mean acceleration as explanatory variables, and individuals as a random factor, for each species. Movement distance and mean acceleration were treated as covariates because these variables may be independently correlated with the onset of bill-grasping. Individual birds were included as random effects. We did not consider velocity, because the velocity and movement distance were correlated with each other, and unreliable regression results could occur if we used both parameters in the analysis of one model (electronic supplementary material, figure S1). Significance of explanatory variables was evaluated by a likelihood ratio test with the 5% level. Confidence intervals of estimated parameters for the timing of grasping onset were used to compare the effects of the bill extension between the experimental phases. The goodness of fit of the linear model was assessed by marginal and conditional *R*^2^ values. All of these analyses were performed using R v. 3.1.2. [26] with packages ‘lme4’ for the linear mixed model [27], ‘car’ for the likelihood ratio test [28] and ‘MuMIn’ for marginal and conditional *R*^2^ values [29,30].

## 3. Results

### 3.1. Pigeons

A total of 974 instances of pecking were recorded from the three pigeons: 118 instances in the control, 49 in session (S)1, 221 in S2–4, 218 in S5–7, 231 in S8–10, 84 in bill-extension removal and 53 in the follow-up.

**Figure 3.**
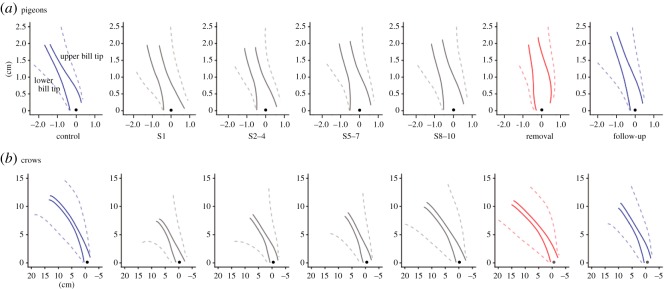
Movement trajectories reconstructed by the tracking markers on the tips of original upper and lower bills during pecking of pigeons (*a*) and crows (*b*) across the phases. Two solid lines in each panel show the mean trajectories of marker-tracked upper and lower tips of the bill. *X*–*Y* axis represents the relative distance of the marker-tracked bill tips to the target food as the origin. Dashed lines represent 1 s.d. A filled small dot at the bottom of the panel indicates the target food.

Movement trajectories of upper and lower bills during pecking are illustrated in figure 3*a*. Normal pecking without bill extensions in the control phase showed that the grasping aperture increased while approaching food and decreased just before contact with food (figure 3*a*, control; also see electronic supplementary material, movie P1). After attachment of the bill extension in S1, no clear decrease in the grasping aperture appeared before contact with food (figure 3*a*, S1; also see electronic supplementary material, movie P2), indicating that pigeons did not compensate by decreasing their grasping aperture soon after their bills were extended. There was no decrease in the grasping aperture before contact with food and the movement trajectories similar to that in S1 were kept through the bill-extension phases (figure 3*a*, S2–4 to S8–10). Conversely, in the bill-extension removal phase, the decrease in the grasping aperture occurred too early before contact with food in comparison to the control phase (figure 3*a*, removal; also see electronic supplementary material, movie P3). The disappearance of the premature opening in the follow-up phase (figure 3*a*, follow-up) indicated recovery to normal pecking after one week without the bill extension.

To verify whether pecking performance and kinematics could be influenced by the position of target and order of pecks in a series at an array of seeds, mixed-model analyses were employed for normal pecking behaviour using the data from the control phase. For pecking performance, we executed a binomial mixed-model analysis that included grasping success (i.e. success or failure for each peck) as the dependent variable; ‘target position’, ‘order’ in a series of pecks, and their interaction as independent variables; and individuals as a random factor. The output of model analysis revealed no significant effect of any independent variable (target position, $\chi_{1}^{2}=0.01$, *p* = 0.91, n.s.; number, $\chi_{1}^{2}=0.07$, *p* = 0.79, n.s.; target position × number, $\chi_{1}^{2}=2.07$, *p* = 0.15, n.s.; electronic supplementary material, figure S2), indicating no effect of the position of the target or the order of pecks in a series on performance. For grasping onset of pecking kinematics, we executed a linear mixed-model analysis that included grasping onset as the dependent variable; target position, order of pecks in a series and their interaction as the independent variables; and the individual as a random factor. The output of the model analysis failed to reveal significant effects of any independent variable (position, $\chi_{1}^{2}=0.03$, *p* = 0.99, n.s.; number, $\chi_{1}^{2}=0.09$, *p* = 0.76, n.s.; position × number, $\chi_{1}^{2}=0.02$, *p* = 0.88, n.s.; electronic supplementary material, figure 3S3a,b). In addition, a similar mixed-model analysis was conducted for the movement distance but, again, there were no effects of any independent variable (target position, $\chi_{1}^{2}=1.55$, *p* = 0.21, n.s.; pecking order $\chi_{1}^{2}=1.95$, *p* = 0.16, n.s.; target position × pecking order = 0.0001, *p* = 0.99, n.s.; electronic supplementary material, figure 3S3*c*,*d*). These statistical results indicated no effect for the order and target position factors of successive pecks on both grasping onset and movement distance for each peck. Based on these results, we were able to treat successive pecks at an array of seeds as independent events.

**Figure 4.**
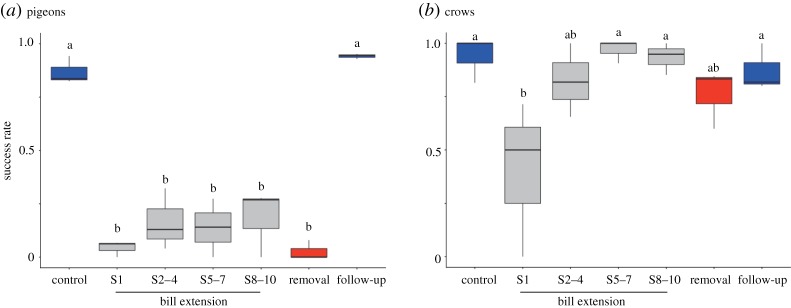
Success rates for ingesting foods across the phases in pigeons (*a*) and crows (*b*). Different letters above the box plots indicate a significant difference from the control phase based on 95% CI (see electronic supplementary material, table S1 for statistics).

The success rate of ingesting foods severely decreased after the attachment of bill extensions and immediately after the removal of the bill extension (figure 4*a*). Mixed-model analyses on the success rate revealed the significant effect of the session variable ($\chi_{7}^{2}=189.41$, *p* < 0.001, overdispersion parameter = 1.26). Comparisons of 95% CIs showed that success rates decreased drastically in all the bill-extension sessions compared with successes rates in the control phase (figure 4*a*; see electronic supplementary material, table S1 for individual data), indicating that no improvement of pecking success occurred at the performance level during the bill-extension sessions. Interestingly, poor pecking performance continued immediately after the removal of the bill extension, supporting the possibility that a motor adaptation to the bill extension implicitly occurred during the bill-extension sessions.

**Figure 5.**
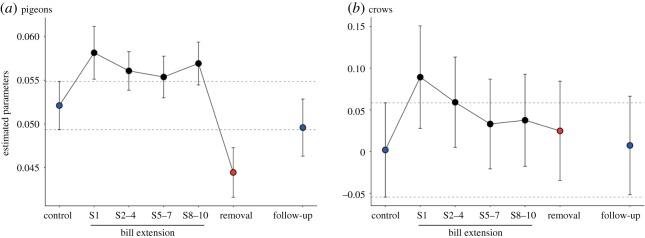
Estimated parameters by mixed-model analyses for grasping onset across the phases while controlling the effects of movement distance and mean acceleration variables. Error bars represent 95% CIs of the parameters. Dashed lines denote the range of 95% CIs of the control phase.

At the kinematic level, pecking motor adaptation to the extended bill was confirmed because the effects on grasping onset continued across the sessions. Linear mixed-model analysis for grasping onset revealed the significant effects of phase ($\chi_{7}^{2}=2927.26$, *p* < 0.001), movement distance ($\chi_{1}^{2}=1991.59$, *p* < 0.001) and mean acceleration ($\chi_{1}^{2}=1542.40$, *p* < 0.001). As shown in figure 5*a*, we found that estimated parameters for grasping onset shifted towards positive values immediately after the attachment of the bill extension in S1, indicating a delay in the grasping onset because of attachment of the bill extension. This temporal delay indicated that pigeons failed to start to close the extended bills earlier than they did the original bills, in order to compensate for the shortened distance to the target caused by the extended length of the bills. The temporarily delayed shift of grasping onset appeared to remain across all bill-extension phases (S2–4 to S8–10), although their confidence intervals were not clearly different from those of the control phase. By contrast, the estimated parameter in the bill-extension removal phase was found to shift towards more negative values in relation to that in the control phase, indicating the temporal advancement of grasping onset immediately after removing the bill extension. More specifically, immediately after the removal of the bill extension, pigeons without bill extension started to open their bills earlier than they did in the control phase. This temporal advancement of grasping onset suggests a pecking motor adjustment to compensate the temporal delay of grasping onset by the bill extension. However, the temporary advancement of grasping onset in the bill-removal phase disappeared in the follow-up phase, indicating that grasping onset was recovered to normal after one week with a normal bill.

**Figure 6.**
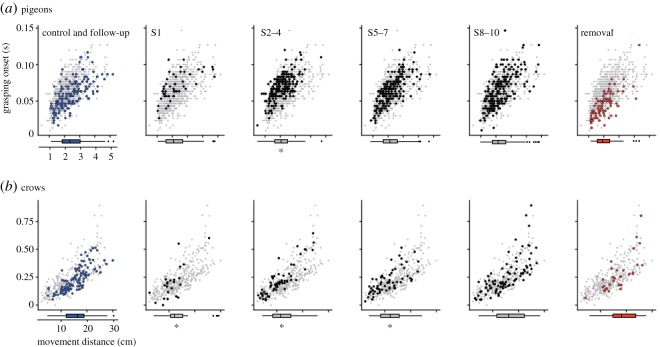
Scatter plots of bill-grasping onset and movement distance of head-reaching in pigeons (*a*) and crows (*b*). Box plots below the horizontal axis denote the distributions of movement distances for comparison between phases. Note that the data for the control and follow-up were combined into one panel because no significance was found between the two phases. Asterisks under the bar plot depict a significant difference from the control and follow-up based on 95% CIs. The pale grey plots in the background of each panel show the entire distribution of the plots.

**Figure 7.**
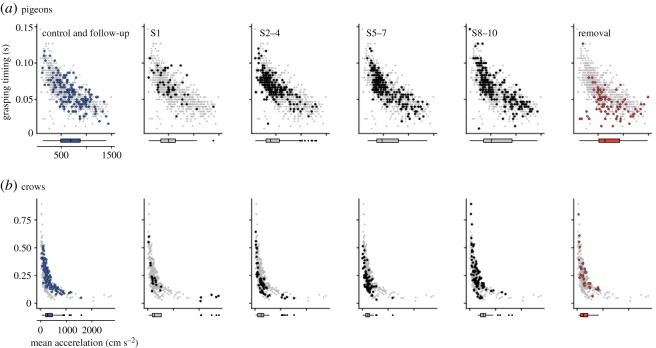
Scatter plots of the bill-grasping onset and mean acceleration of head-reaching in pigeons (*a*) and crows (*b*). Box plots below the horizontal axis denote the distributions of movement distances for comparison between phases. Note that the data of the control and the follow-up were combined into one panel because no significance was found between the two phases. The pale grey plots in the background of each panel show the entire distribution of the plots.

There was no clear difference in both movement distance and mean acceleration of head-reaching across the phases (figures 6*a*, 7*a*). In S2–4, a shorter movement distance than in the control was detected. However, the effects on movement distance and mean acceleration of head-reaching because of the bill extension were scarce when compared with grasping onset.

### 3.2. Crows

A total of 378 pecking events were recorded for the three crows: 80 instances in the control, 23 in S1, 61 in S2–4, 65 in S5–7, 88 in S8–10, 30 in bill-extension removal and 31 in the follow-up.

Bill trajectories during pecking in crows are illustrated in figure 3*b*. No decrease in grasping aperture immediately before contact with food can be seen in S1, although the effect was not as large as was seen for pigeons with bill extension (figure 3*b*, S1; also see electronic supplementary material, movies C1 and C2). However, trajectories in the following bill-extension phases appeared similar to that observed in the control (figure 3*b*, S1 to S8–10). In contrast with pigeons, an excessively early decrease of grasping aperture could not be seen immediately after the removal of the bill extension, but rather the trajectory appeared similar to that in the control, as well as in the follow-up (figure 3*b*, removal and follow-up; also see electronic supplementary material, movie C3), suggesting rapid adjustment of pecking to both the attachment and removal of the bill extensions.

Success rates of ingesting food decreased transiently after the attachment of the bill extension in S1 (figure 4*b*). Mixed-model analyses revealed the significant effect of the phase variable ($\chi_{7}^{2}=53.52,$ *p* < 0.001, overdispersion parameter = 1.10). Comparisons of 95% CIs showed a significant decrease in success rates only in S1, but not in any other phases, compared with that observed in the control phase (figure 4*b*; see electronic supplementary material, table S1 for individual data). These results indicated that pecking performance diminished following the attachment of the bill extension transiently, but this did not last for a long time or occur after its removal, suggesting rapid adjustment of the pecking to the bill extension and its removal.

At the level of grasping onset, the effects of the bill extension and its removal were much less than those found in pigeons. Linear mixed-model analysis for grasping onset detected the significant effects of phase ($\chi_{7}^{2}=44.98$, *p* < 0.001), movement distance ($\chi_{1}^{2}=611.86$, *p* < 0.001) and mean acceleration ($\chi_{1}^{2}=86.15$, *p* < 0.001). Comparison of 95% CIs of estimated parameters showed no significant difference among any phases, suggesting that crows quickly adjusted their onset of grasping to the bill extension and its removal without motor adaptation as found in pigeons (figure 5*b*).

Interestingly, we found a significant difference in the movement distance in S1, S2–4 and S5–7 from that observed in the control (figure 6*b*). Conversely, there was no difference in mean acceleration among any phases (figure 7*b*). These results indicated that crows pecked the food at the closer distance, soon after the attachment of the bill extension, than was observed in the control.

## 4. Discussion

In this study, we examined motor control flexibility in the adjustment of pecking with an artificial extension of the bill comparatively in pigeons and crows. We confirmed that pigeons and crows both possess the plasticity to adjust their pecking to bill extension, but were remarkably different in the degree of flexibility. Pigeons showed a clear deterioration in grasping foods with the bill extension, without improvement after 160–300 pecking experiences with the extension. However, after the removal of the bill extension, a motor after-effect, observed as a temporarily advanced grasping onset, suggested the presence of a motor plasticity to the bill extension. In contrast with pigeons, crows showed deterioration in grasping foods only transiently after the bill extension was attached, and recovered rapidly to normal pecking levels. In addition, no after-effect emerged after bill-extension removal. These contrasting results from the two species suggest different visuomotor mechanisms of pecking control between pigeons and crows. Our findings are the first empirical evidence of the plasticity in pecking control in birds and, in particular, the flexible capacity of rapid adjustment to a physically extended part of the bill in crows.

The difference in flexibility of pecking movements in response to the bill extension between pigeons and crows found in this study might have been influenced by the different materials used for target foods provided to the two species. Cheese presented for crows is softer and, in general, might be easier to grasp than hempseed for pigeons. This qualitative difference in target foods might make it more advantageous for crows to successfully grasp the foods by the resin bill extension than for pigeons. In fact, the success rate of food ingestion dropped more in pigeons than in crows. However, the clear decrease in the success rate of food ingestion in the crows in the first session with the bill extension indicated that the bill extension did perturb their pecking. This deteriorative effect in response to the bill extension in crows indicated that food quality for grasping was not so advantageous for crows that it cancelled out the perturbation effects of the bill extension.

There is a possibility that pecking a piece of cheese was easier for crows than pecking seed from an array was for pigeons because pigeons needed to move every time between different positions to peck. However, this possibility is unlikely for three reasons. First, during the control phase, as shown in figure 4, pigeons and crows showed comparable levels of performance, approximately 90% success, suggesting there was no difference in the level of difficulty of pecking targets between the two species. Second, all pecks analysed in this study were confirmed to execute after a short stop (i.e. head fixation) in both pigeons and crows, indicating that successive pecks of pigeons were treated as independent events comparable with single-shot pecks of crow. Third, as seen in the plots in figure 6, the movement distance in crows was varied between pecks even at the same locations of the target. This was true for pigeons. The mixed-model analyses of pigeon pecks in the control phase revealed no significant effects of the target position on both pecking performance and kinematic parameters, such as movement distance and grasping onset. These statistical results suggested that the distance to a seed at the same location was still varied between peck events in pigeons. Therefore, it is unlikely that the different manner of target presentation between pigeons and crows was advantageous for either species and caused the differences in the difficulty observed in pecking the target(s).

The contrasting performance in pecking adjustment to bill extensions between pigeons and crows could be explained by the difference in the bill-extension lengths. The bill-extension lengths relative to the original bills of pigeons were slightly longer than those of crows. Longer bill extensions for the pigeons might have been responsible for their poorer pecking adjustment to the extended bills in comparison to that of crows. However, immediately after the bill extension in S1, pecking performance significantly deteriorated in both pigeons and crows. This indicated that the bill extension was effective in pecking control not only in pigeons but also in crows. In addition, if differences of the bill-extension length primarily influenced pecking adjustment to the extended bill, then pecking performance and kinematics would not be in contrast as they were in the present results, but rather would be quantitatively different between pigeons and crows. Specifically, pecking performance of pigeons would be improved through the number of pecking experiences, which was higher for pigeons that pecked arrays of seeds than for crows that pecked small pieces of cheese in our experimental setting. Nevertheless, no clear improvement occurred in pecking performance in pigeons in contrast with the rapid improvement of crows. Although there was a small effect caused by the difference of relative bill-extension lengths between pigeons and crows on pecking, it is unlikely that such difference primarily caused the contrasting results of motor adaptation between the two species.

Pecking experiences outside the experimental situation, such as feeding in home cages, were unlikely to cause contrasting results between pigeons and crows. Both pigeons and crows were allowed to feed from cups filled with foods in their home cages. Feeding from food-filled cups was easy for both pigeons and crows even with bill extensions, because no accurate control of pecking at a specific target, such as occurred in the experiment, was necessary to ingest foods, but rather just the bill-opening action into the cups was sufficient to take foods into the mouth. Indeed, both pigeons and crows were observed to ingest foods from the cups without any difficulty, irrespectively of the bill extension. Thus, feeding in the home cages did not cause critical differences of bill-use experiences that could explain the contrasting results between pigeons and crows.

The quantitative difference in pecking experiences with bill extensions between the two species might have facilitated the contrasting results; however, this is unlikely. In this study, we introduced fewer pecking opportunities to crows than pigeons because of the limited motivation of crows to peck the target under the experimental setting. Fewer pecking experiences with the bill extension in crows might have inhibited their motor adaptation in comparison to pigeons, resulting in the absence of a motor after-effect in crows. However, we should emphasize that the main goal of our present study was not to examine the motor adaptation ability *per se* but to compare the flexibility of pecking adjustment to the bill extension between the two species. Of importance in the present results is the rapid adjustment of pecking after the clear perturbation by the bill extension in crows in comparison to pigeons. The contrasting results found in this study are unlikely to be explained as artefacts caused by the difference in food materials and/or number of pecking experiences with the bill extension, but suggest that the different mechanisms underlay motor control of pecking in response to the bill extension.

The results from pigeons were consistent with the previous findings of pecking control mechanisms in pigeons. Pecking by pigeons has been suggested to be controlled feed-forwardly according to a preplanned movement in the fixation period without online visual feedback for ongoing movement correction [6,7,9,11]. If online visual feedback is involved in pecking, kinematic parameters, such as bill-grasping onset, should be maintained or soon recovered to the similar level of pecking with a normal bill even after the attachment of the bill extension. However, this was not the case in our study. Instead, the results from pigeons appeared consistent with the feed-forward control mechanism. If the feed-forward control merely operates pecking by pigeons, it is expected that pecking would be persistent following bill-extension attachment, and could not quickly change because no online movement correction could be used to adjust the movement. The only available feedback information to adjust pecking movement could be the somatosensory signals regarding physical contact of the bill tip with the target. By the attachment of the bill extension, the timing for the bill tip to contact the target would be temporarily advanced because the movement distance was shortened correspondingly to the extended length of the bill. Thus, in the early phases with the bill extension, an error would be produced between the actual timing of bill contact with the target and the predicted timing, which is calculated from the visually perceived distance to the target according to motor programs based on pecking experiences with the normal bill. This temporal error might be useful as a feedback signal to temporarily advance the grasping onset to contact the extended bill with the target in appropriate time. Although the involvement of somatosensory feedback from the bill for pecking adjustment is beyond the scope of the present study, the results for pigeons in both movement trajectory and the motor after-effect in grasping onset suggested that pecking of pigeons was controlled by feed-forward and offline feedback mechanisms.

Quick adjustment of pecking to the bill extension in crows supports the possibility of online visually guided control. Deterioration of food ingestion in the first session of the bill-extension phase indicated that the artificial bill actually perturbed pecking of crows. This effect can be observed in the slight overshooting of the bill-movement trajectory and partly temporal delay of bill-grasping onset in the first bill-extension session. However, all of these effects disappeared in the next phase, S2–4, indicating that the crows adjusted pecking to the bill extension. Furthermore, after 10 sessions with the bill extension, its removal produced no after-effect. In addition, crows kept open their eyes during pecking; by contrast, pigeons often closed their eyes during pecking (see electronic supplementary material, movies C1–3 and P1–3). These results suggest that the crows were able to quickly adjust pecking not based on such error-driven motor learning as was suggested in pigeons, but rather on a more flexible visuomotor mechanism.

Interestingly, we found a significant decrease of the movement distance during the bill-extension phases, such as S1, S2–4 and S5–7, in comparison to that of the control phase. This indicated that the crows initiated head-reaching in closer range to the target after bill extension. Given the covariation between the movement distance and the mean velocity of head-reaching (see the §2.5. Analysis, in Materials and methods; also see electronic supplementary material, figure S1), it is likely that the crows adjusted pecking to the bill extension with slower velocity from a closer distance to the target, although we did not determine which parameter was crucial for pecking adjustment in crows. In either case, taking into account the speed–accuracy trade-off in human arm-reaching [31,32], pecking with slower velocity in closer range could be an effective solution to execute head-reaching and bill-grasping accurately to the target with the bill extension, otherwise grasping the target would have failed. Although our present study did not reveal the actual involvement of online visual feedback to adjust pecking in crows, such as arm-reaching in humans, the quick modification of pecking parameters such as velocity and/or distance after one session of experience, suggested the flexible visuomotor capacity of crows to control the head/bill movement appropriately to the extended body part.

In conclusion, our present study revealed that crows are capable of rapid motor adjustment of pecking to bill extension, but pigeons are not, although they possessed plasticity in a form of motor adaptation. These contrasting results of sensorimotor flexibility to body-part extension suggest different mechanisms underpinning pecking control between pigeons and crows: feed-forward and offline feedback control in pigeons and, presumably, online feedback control in crows. These differences in pecking control mechanisms might be related to their foraging behaviour. Less plastic pecking in pigeons is optimal and even efficient to feed on static seeds and grains on the ground. On the other hand, flexible head-reaching and bill-grasping in crows might be crucial for omnivorous/carnivorous foraging to catch moving targets, such as insects and small vertebrates. Similar motor flexibility of omnivorous/carnivorous birds was reported in Australian ravens (*C. coronoides*), compared to pigeons and small passerines, in a recent study, suggesting the close relevance of motor flexibility to innovative foraging behaviour [24]. Similar relationships between flexible motor control and innovative foraging behaviour might be applicable in omnivorous/carnivorous mammals [33], particularly in tool use of human and non-human primates [4,34,35]. However, there is the possibility that the flexible motor control in birds might be associated, at the ultimate mechanism level, with nest building [36,37]. Proximate mechanisms of sensorimotor flexibility for dextrous motor control such as seen in crows are still open to questions. The sensory feedback signals and how they operate in online control, such as fast reaching within 100–150 ms in crows, require investigation. Also, it is necessary to investigate what ‘body schema’ [34] is represented and incorporated to the extended bill in the large multisensory pallium of crows [38,39]. These lines of future psychophysical and neurophysiological studies would be needed to understand the different or similar mechanisms underlying the analogously dextrous foraging skills, such as tool use, between the animals with different body structures.

## Supplementary Material

Figure S1>Plots of mean velocity and movement distance

## Supplementary Material

Figure S2>Success rates at different position in an array and the order of successive pecks in the control phase.

## Supplementary Material

Figure S3>Grasping onset at different positions in an array and the order of successive pecks in the control phase.

## Supplementary Material

Table S1>Individual behavioural data

## Supplementary Material

Data S1>Raw data

## Data accessibility

Raw data are available as the electronic supplementary material.
