# Supplementary figures and images for: Flexible motor adjustment of pecking with an artificially extended bill in crows but not in pigeons
Source: R Soc Open Sci. 2017 Feb 15;4(2):160796. doi: 10.1098/rsos.160796 (PMC5367294; doi:10.1098/rsos.160796)

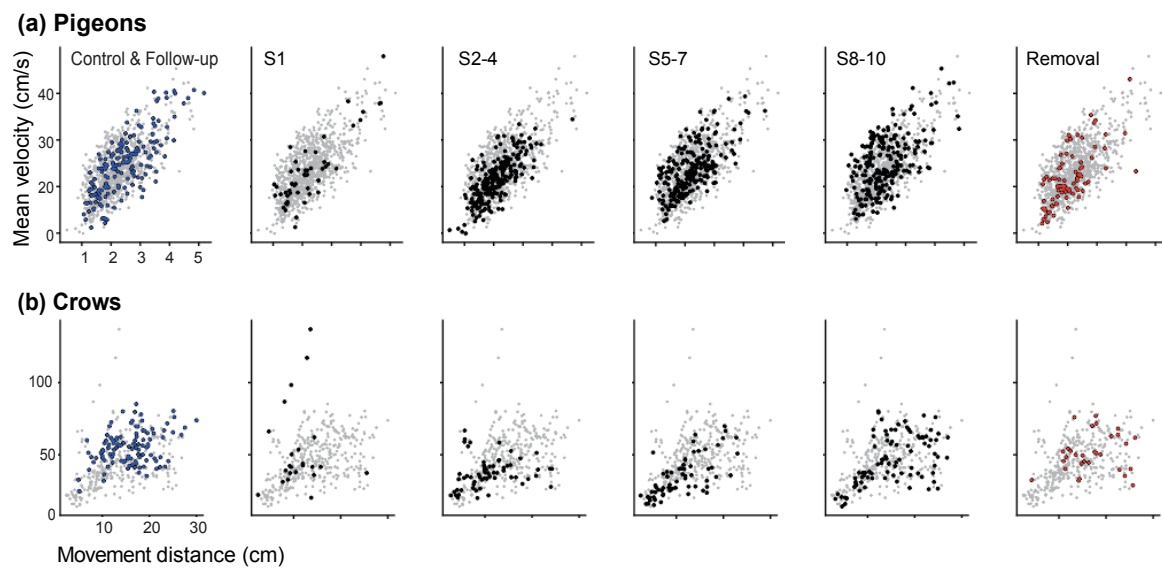

Supplement: Figure S1>Plots of mean velocity and movement distance [file rsos160796supp1.pdf]

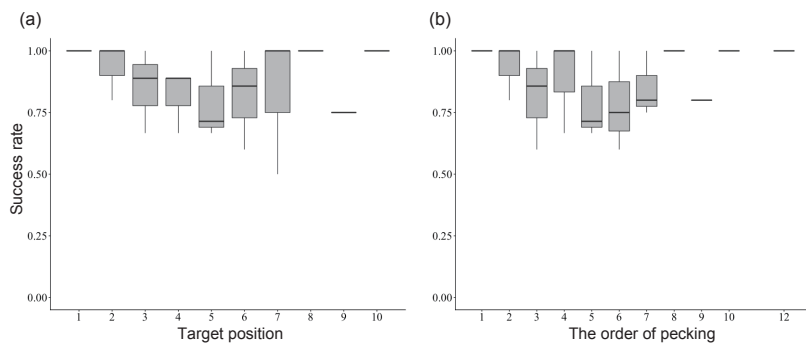

Supplement: Figure S2>Success rates at different position in an array and the order of successive pecks in the control phase. [file rsos160796supp2.pdf]

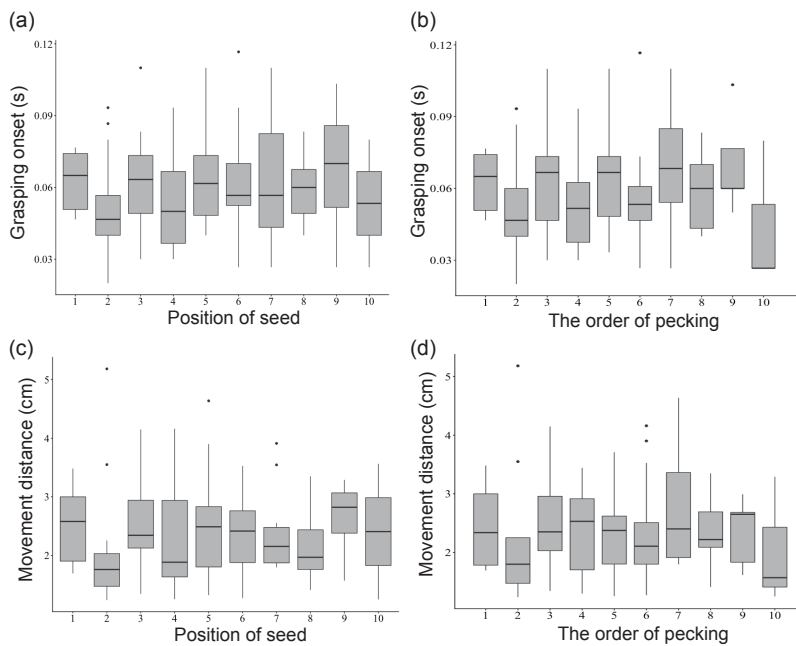

Supplement: Figure S3>Grasping onset at different positions in an array and the order of successive pecks in the control phase. [file rsos160796supp3.pdf]
