# Supplementary material for: Flexible motor adjustment of pecking with an artificially extended bill in crows but not in pigeons
Source: R Soc Open Sci. 2017 Feb 15;4(2):160796. doi: 10.1098/rsos.160796 (PMC5367294; doi:10.1098/rsos.160796)
Supplement: Table S1>Individual behavioural data [file rsos160796supp4.pdf]

Supplementary Table 1. The numbers of success (/pecks in each session) to ingest foods in each subject and 95% confidence intervals.

Experimental Phase		Pigeon#1	Pigeon#2	Pigeon#3	Crow#1	Crow#2	Crow#3
Control		36/43	33/35	33/40	28/28	25/25	22/27
			(1.070, 3.246)			(0.025, 0.945)	
Bill extension	S1	0/2	2/32	1/15	3/6	5/7	0/10
			(-4.881, -1.870)			(1.661, 4.473)	
	S2−4	3/74	30/93	7/54	9/11	21/21	19/29
			(-2.769, -0.774)			(-1.997, 0.793)	
	S5−7	0/41	29/106	10/71	21/21	12/12	29/32
			(-2.955, -0.950)			(2.052, 5.247)	
	S8−10	0/55	23/83	25/93	22/22	37/39	23/27
			(-2.618, -0.642)			(1.515, 4.224)	
Bill extension removal		0/25	2/34	2/25	3/5	11/13	10/12
			(-5.748, -2.369)			(0.260, 3.105)	
Follow-up		20/21	13/14	17/18	9/11	10/10	8/10
			(1.710, 4.751)			(0.677, 3.705)	

Values in the parenthesis indicate the range (2.5%, 97.5%) of confidence intervals of estimated parameters from the mixed model analyses. Note the number of pecks were different among sessions of each subject because of terminating the session due to no response by subject and/or video-recording failure.
